# Identifying mouse developmental essential genes using machine learning

**DOI:** 10.1242/dmm.034546

**Published:** 2018-12-13

**Authors:** David Tian, Stephanie Wenlock, Mitra Kabir, George Tzotzos, Andrew J. Doig, Kathryn E. Hentges

**Affiliations:** 1 Division of Evolution and Genomic Sciences, Faculty of Biology, Medicine and Health, Manchester Academic Health Science Centre, The University of Manchester, Oxford Road, Manchester M13 9PT, UK; 2 Department of Agriculture, Food and Environmental Sciences, Marche Polytechnic University, Ancona 60121, Italy; 3 Manchester Institute of Biotechnology, The University of Manchester, 131 Princess Street, Manchester M1 7DN, UK; 4 Division of Neuroscience and Experimental Psychology, Faculty of Biology, Medicine and Health, The University of Manchester, Manchester M13 9PT, UK

**Keywords:** Essential genes, Supervised machine learning, Mouse knockout, Essentiality database

## Abstract

The genes that are required for organismal survival are annotated as ‘essential genes’. Identifying all the essential genes of an animal species can reveal critical functions that are needed during the development of the organism. To inform studies on mouse development, we developed a supervised machine learning classifier based on phenotype data from mouse knockout experiments. We used this classifier to predict the essentiality of mouse genes lacking experimental data. Validation of our predictions against a blind test set of recent mouse knockout experimental data indicated a high level of accuracy (>80%). We also validated our predictions for other mouse mutagenesis methodologies, demonstrating that the predictions are accurate for lethal phenotypes isolated in random chemical mutagenesis screens and embryonic stem cell screens. The biological functions that are enriched in essential and non-essential genes have been identified, showing that essential genes tend to encode intracellular proteins that interact with nucleic acids. The genome distribution of predicted essential and non-essential genes was analysed, demonstrating that the density of essential genes varies throughout the genome. A comparison with human essential and non-essential genes was performed, revealing conservation between human and mouse gene essentiality status. Our genome-wide predictions of mouse essential genes will be of value for the planning of mouse knockout experiments and phenotyping assays, for understanding the functional processes required during mouse development, and for the prioritisation of disease candidate genes identified in human genome and exome sequence datasets.

## Abstract (precis)

**Summary:** Here, we used computer-based machine learning methodology to predict which genes in the mouse genome are essential for development, and present a database of mouse essential and non-essential genes.

## INTRODUCTION

Essential genes are those that are required for the survival of an organism. Although studies in unicellular organisms, such as yeast, have experimentally defined the set of essential genes in those species (Kofoed et al., 2015), the large genome size and developmental complexity of animal models have hindered a comprehensive experimental essentiality analysis in these organisms. Knowledge of essential genes in animal species is informative for understanding the biological functions required during development, as well as for identifying candidate genes for human genetic diseases. In particular, the mouse has been a long-standing model for human disease research due to the ability to generate specific genome alterations in mouse embryonic stem cells, allowing the targeted deletion or knockout of individual genes. Mouse knockout experiments have proved useful in identifying a subset of mammalian essential genes (Sung et al., 2012); however, the entirety of the mouse genome has not yet been experimentally examined.

Current efforts to experimentally investigate gene function using mouse models are enhanced by the creation of the International Knockout Mouse Consortium (IKMC) (Bradley et al., 2012), a large global project with the goal of generating knockouts for over 20,000 protein-coding mouse genes. The International Mouse Phenotyping Consortium (IMPC) (Ayadi et al., 2012; Brown and Moore, 2012) builds upon the efforts of IKMC to discover functional insights for every gene by systematically phenotyping over 20,000 knockout mouse strains. In order to optimise knockout experiment design, machine learning algorithms (Yuan et al., 2012) have been used to predict the essentialities of mouse genes based on their genomic features. Moreover, predicting the essentialities of mouse genes using machine learning algorithms can aid in the identification of candidate genes for human genetic diseases, due to the close genetic and physiological similarities between mouse and human (Rosenthal and Brown, 2007). Machine learning methods are also useful in identifying features associated with gene essentiality (Kabir et al., 2017).

A variety of machine learning methodologies have proven useful in predicting essential genes in several organisms. Many studies have sought to identify bacterial and fungal essential genes, because knowledge of gene essentiality in microbial species can reveal potential drug targets (Yu et al., 2017; Hua et al., 2016; Deng, 2015; Ning et al., 2014; Lu et al., 2014; Cheng et al., 2014; Cheng et al., 2013; Deng et al., 2011; Plaimas et al., 2010; Seringhaus et al., 2006; Gustafson et al., 2006; Liu et al., 2017; Nigatu et al., 2017). *Saccharomyces* *cerevisiae* essential genes have been identified using machine learning classifiers trained on multiple characteristics of protein function, such as physical, metabolic and transcriptional regulatory interactions, gene expression patterns and annotated biological functions (Acencio and Lemke, 2009; Zhong et al., 2013; Hwang et al., 2009). Protein interaction network topologies have also been utilised for the prediction of human essential genes (Yang et al., 2014). The lack of functional annotation of the majority of plant genes, and the long generation time required for experimental analysis of mutant plant phenotypes, provided the motivation to implement a random forest machine learning algorithm for the prediction of *Arabidopsis thaliana* essential genes (Lloyd et al., 2015); similar challenges underlie the identification of mammalian essential genes.

In order to provide insights into the gene functions required during mammalian development, we identified a dataset of genes needed for a mouse embryo to survive until the postnatal period, which we define as essential genes (Kabir et al., 2017). Here, we implement a supervised machine learning approach to generate an essentiality classifier, testing a variety of machine learning methods. We found that random forests provided the most accurate classifier and, following feature selection, achieved classification accuracy of greater than 95% during 10-fold cross-validation. The accuracy of our classifier was also assessed against 2 blind test sets, and over 80% accuracy was achieved on these datasets. The classifier was then used to predict the essentiality of the remaining protein-coding genes in the mouse genome. Functions linked to each essentiality class were identified, and the transferability of our classifications was determined by comparing our predictions with experimental data from mouse mutants generated through non-knockout experimental methods and human gene essentiality annotations. We conclude that our predictions have a high degree of accuracy, and thus could facilitate mouse knockout experimental design and contribute to a deeper understanding of biological functions that are essential for mammalian development.

## RESULTS

### Training and test sets

Manually curated datasets containing 1307 essential genes (those with pre- or perinatal lethal phenotypes in mouse knockout experiments) and 3459 non-essential genes (those with viable phenotypes in mouse knockout experiments) (Kabir et al., 2017) were used as the input to our classifier. In total, 102 features (Tables S1 and S2) were identified from multiple public databases as characteristics that might distinguish between essential and non-essential genes. In total, 75 of the 102 features analysed had statistically significant differences in values between genes in the essential and non-essential training sets (Kabir et al., 2017). Owing to the large number of features with distinct values, we hypothesised that essential and non-essential genes could be differentiated by their properties. We therefore sought to test a variety of machine learning methods to identify the most accurate approach to categorise genes as essential or non-essential. Our original dataset is an imbalanced dataset as the number of non-essential genes is much larger than the number of essential genes. Imbalanced datasets can degrade the classification performance of machine learning classifiers due to their bias towards classifying instances belonging to the majority class (Visa and Ralescu, 2005). Therefore, to develop a machine learning classifier, we generated balanced training sets containing all 1307 essential genes, and 1307 non-essential genes selected at random from the total set of 3459 non-essential genes (Table S3). To remove possible bias, this process was repeated 10 times in order to generate 10 different balanced training datasets containing different sets of non-essential mouse genes (Table S3). We further developed 10 random forest classifiers by implementing 10-fold cross-validation on these training datasets, utilising all features. We found a very small range in the cross-validation accuracies (89.89-91.42%) (Table S4), showing that the choice of genes in the training datasets had little effect. The mean accuracy of these classifiers was 90.90%; therefore, we selected the training dataset that had an accuracy of 90.85% for all further experiments, as this was closest to this mean value. We might have overestimated the overall performance of our classifier if we selected a training dataset for which the cross-validation accuracy was more than the mean value.

In order to evaluate the accuracy of the machine learning classifiers, we assembled test sets. Test set 1 (Table S3) contained 229 essential and 802 non-essential genes, the essentiality status of which was published by the IMPC either in the literature or via their website (Koscielny et al., 2013) after our training sets were compiled. Test set 2 (Table S3) was formed of the 2152 genes in our original non-essential gene dataset that were not incorporated into the balanced essential and non-essential training sets. Test set 4 contained 169 lethal and 441 viable genes, which were added to the IMPC database at the conclusion of the project (April 2018), and were not already included in our training datasets or in Test sets 1 and 2. Test set genes were not used in classifier training.

We also compiled a prediction dataset containing all genes in the mouse genome with no experimental essentiality annotations (Test set 3). MouseMine (Motenko et al., 2015) was used to retrieve all known mouse genes. In total, 22,944 protein-coding mouse genes were identified. After excluding genes with known essentiality that are included in training and test sets, and removing non-mouse genes and duplicate gene names from the MouseMine dataset, 15,495 unique protein-coding genes with unknown essentiality status remained in Test set 3 (Table S3). All the features previously collected for training set genes were then collected for test set genes, following the same methodology used for compiling training set features (Kabir et al., 2017).

### Data pre-processing

We found that there were no data available for several features for genes in the training and test datasets. We found that 10 features of the protein-protein interaction (PPI) network compiled from known PPIs had missing values for nearly 40% of the genes in the training set, so these features (Table S2) were removed from classifier training. The other 92 features had missing values for fewer than 12% of the genes. For classifier training, the missing values of these features were replaced with the feature mean values. Following the replacement of missing values, features within the training datasets were discretised using the ChiMerge algorithm (Kerber, 1992).

### Classifier optimisation

**Fig. 1.**
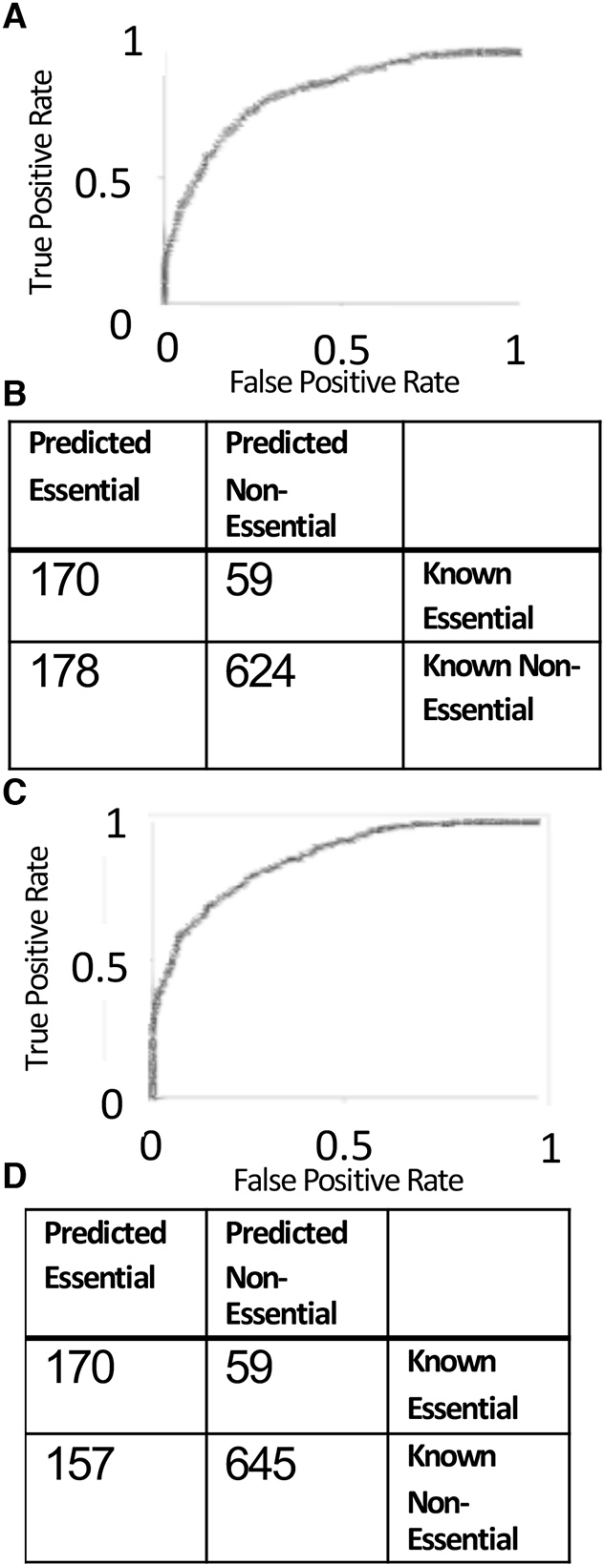
**Prediction accuracies of the random forest classifiers.** Prediction accuracies of the random forest classifiers. (A) ROC plot with AUC 0.803 for the random forest classifier trained on 80 features and tested on Test set 1. (B) Confusion matrix of the random forest classifier trained on 80 features and tested on Test set 1. (C) ROC plot with AUC 0.816 for the random forest classifier trained on the 39 features selected by the genetic algorithm feature selection and tested on blind test set 1. (D) Confusion matrix of the random forest classifier trained on the 39 features selected by the genetic algorithm feature selection and tested on blind test set 1.

**Fig. 2.**
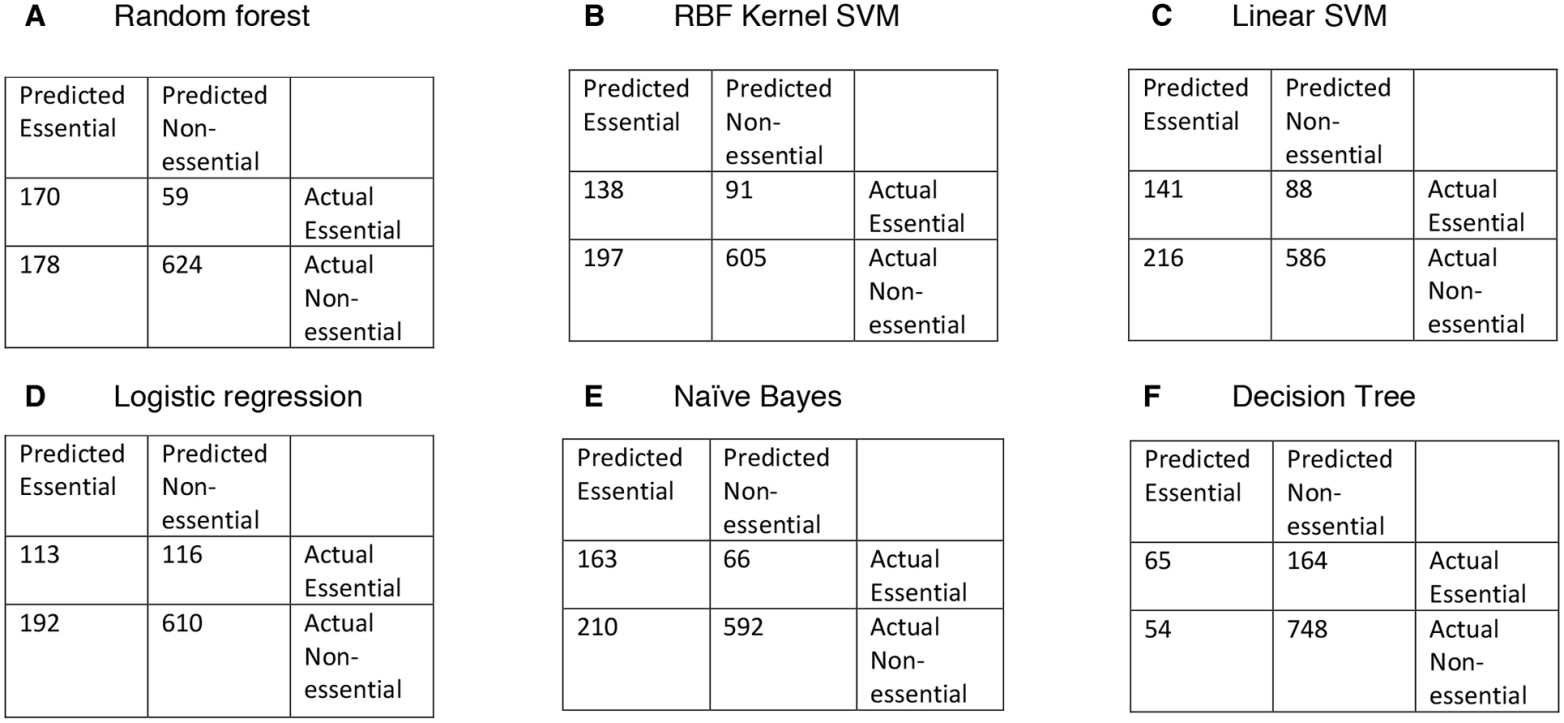
**Confusion matrices of the 6 classifiers trained on all 83 features.** The machine learning algorithm is listed at the top of each chart: (A) random forest; (B) RBF kernel SVM; (C) linear SVM; (D) logistic regression; (E) naïve Bayes; (F) decision tree.

An iterative process was used to test 6 different supervised machine learning classifiers. We assayed random forests, support vector machines (SVMs) with radial basis function (RBF) kernel, polynomial kernel SVMs, logistic regression, naïve Bayes classifier and decision tree classifiers in 10-fold cross-validation on the discretised training sets. We applied information gain feature selection (Yang and Pederson, 1997), and found that 83 features had an information gain greater than 0 (Table S4). These 83 features were ranked in order of significance. Classifiers were tested using increasing numbers of features (ranging from 5 to 83 features) for 10-fold cross-validation on the training sets (Table S4). From these studies, we found that the random forest classifier trained with 80 features had the best performance in 10-fold cross-validation. Using a random forest with 230 trees, we generated a 10-fold cross-validation accuracy of 98.1%. This classifier reached 79.3% accuracy on Test set 1, and the area under the curve (AUC) value of the corresponding receiver operating characteristic (ROC) plot was 0.85 (Fig. 1A). A confusion matrix shows that this classifier predicted 59 known essential genes to have a non-essential function, and 178 known non-essential genes to have essential functions (Fig. 1B). This random forest classifier had an accuracy of 85% on Test set 2. Because Test set 1 contains both essential and non-essential genes, we chose the classifier with the best performance on Test set 1 for further studies. None of the other machine learning methods tested achieved a higher AUC on Test set 1 than the random forest classifier (Fig. 2; Table S4), so the random forest method was used henceforth.

We sought to improve the performance of the random forest classifier by implementing feature selection. When implementing a classifier, an individual feature can be irrelevant, strongly relevant (removal of this reduces the overall prediction accuracy) or weakly relevant (not sufficient alone for prediction). Feature selection, therefore, is a very important stage for the classification problem when using datasets comprised of a large number features, in order to select the most informative features and remove those that simply add noise and thus weaken a predictor. A genetic algorithm (GA) feature selection method (Witten et al., 2011) was applied on the training sets as an alternative method to determine whether a smaller set of features would result in random forests with increased prediction accuracy. The GA found a subset of 39 features (Table S4) after 20 generations that improved the classifier performance. These 39 features belong to 9 types: features of the PPI network representing known PPIs and predicted PPIs, features of the PPI network representing known PPIs only, amino acid content of proteins, gene expression, protein types, subcellular localisation, predicted subcellular localisation and enzyme classes. The PPI network features are ranked highest by information gain, which measures the relevance of a feature, and are the most informative features for predicting the essentiality of protein-coding mouse genes. Notably, features such as gene length, GC content, evolutionary age, presence of transmembrane domains and all Gene Ontology (GO) annotations, which we previously identified as statistically different in their distribution between essential and non-essential genes (Kabir et al., 2017), were not found to improve classifier accuracy and were not incorporated into further classifier training. One reason for this surprising result is that the information in these features could be related to or dependent upon information found in other features, so their inclusion adds no value to the classifier. For example, gene length is not needed if protein length is present.

A random forest classifier was subsequently trained on the 39 features identified from GA feature selection, yielding an improved ROC plot AUC of 0.816 on blind test set 1 (Fig. 1C). The random forest has a true-positive class of 170 instances, true-negative class of 645 instances, false-positive class of 157 instances and false-negative class of 59 instances (Fig. 1D). These results are an improvement over a prior study predicting the essentiality of mouse genes (Yuan et al., 2012). On Test set 2, which only contains non-essential genes, the random forest classifier trained on all 92 features had an accuracy of 80.1%. Following GA feature selection, the random forest classifier trained on 39 features showed an accuracy of 79.9% on Test set 2, showing very little decline in accuracy despite the removal of many features, which allows for increased speed of classification. We formed an additional blind test set of mouse knockout phenotypes published by the IMPC in April 2018 (Test set 4, Table S3). Genes already included in our training sets or Test sets 1 and 2 were excluded from Test set 4. Our random forests classifier trained on 39 features produced accurate predictions for 72% of genes with reported lethal phenotypes and 71% of genes with reported viable phenotypes in Test set 4, consistent with our findings from Test set 1, which included IMPC data reported prior to 2018.

We also compared the overlap between our known essential and non-essential genes obtained from searches of the Mouse Genome Informatics (MGI) database and data released by the IMPC (Koscielny et al., 2013). We found a total of 4752 genes in MGI with essentiality data (Table S4). Of these genes, 3467 have not been tested by the IMPC. In comparing the essentiality annotations for each gene with known essentiality, we did find mismatches between the MGI classifications and IMPC classifications. The percentage of mismatches is greatest for genes classified as essential in MGI and as non-essential by the IMPC. A significant proportion of genes falling into this mismatch category have multiple alleles described in MGI, including both essential and non-essential alleles (owing to experimental differences in gene targeting strategy or strain background); in the IMPC, the phenotype analysis of a single allele has been reported. We calculate that ∼20% of genes with mismatching essentiality status between MGI and the IMPC have variations in the phenotypes produced due to the existence of multiple knockout experiments. Additionally, the IMPC classifies some genes as subviable, defined as genes with knockout alleles whereby homozygous null pups comprise less than 12.5% of a litter (Koscielny et al., 2013), which is a category that we did not include in our essentiality definitions. Of the 432 subviable genes listed in IMPC, 109 are found in our training sets compiled from MGI. Of these 109 genes, ∼20% were contained within our essential gene training set, with the remaining 80% in our non-essential gene sets. Approximately 92% of the subviable genes found within our essential genes training set had additional experimental alleles reported in MGI, which met our definition of essential genes (Table S5). Based on our analysis of the discrepancies between MGI and IMPC data, we predict that as many as 20% of genes will display conflicting essentiality phenotypes depending upon the experimental analysis performed.

### Essentiality predictions

Based on the accurate predictions of genes in Test sets 1 and 2, we used the random forest classifier trained on 39 features (identified from genetic algorithm feature selection) to predict the essentiality status of the remainder of mouse protein-coding genes with no experimental annotations (Table S3). Using this classifier, we found that 28% of genes in the genome are known or predicted essential genes, and 72% of genes in the genome are known or predicted non-essential genes, percentages consistent with mouse knockout experimental results (White et al., 2013; Dickinson et al., 2016). The confidence level for each gene essentiality prediction was determined as a measure of whether or not the prediction is accurate. The confidence level is the fraction of the trees of the random forest that predict an essential gene to be essential, or the fraction of trees that predict a non-essential gene as non-essential. A confidence level of 1 indicates that 100% of trees had the same essentiality status prediction. The confidence levels of the predictions of essential genes are between 0.5 and 0.88, with 1 as the maximum confidence and 0.5 as the minimum confidence. The mean confidence level of essential gene predictions is 0.65. The confidence levels of non-essential gene predictions are between 0.5 and 0.95, with the mean confidence level of non-essential gene predictions being 0.65.

### Applicability to point mutation phenotypes

**Fig. 3.**
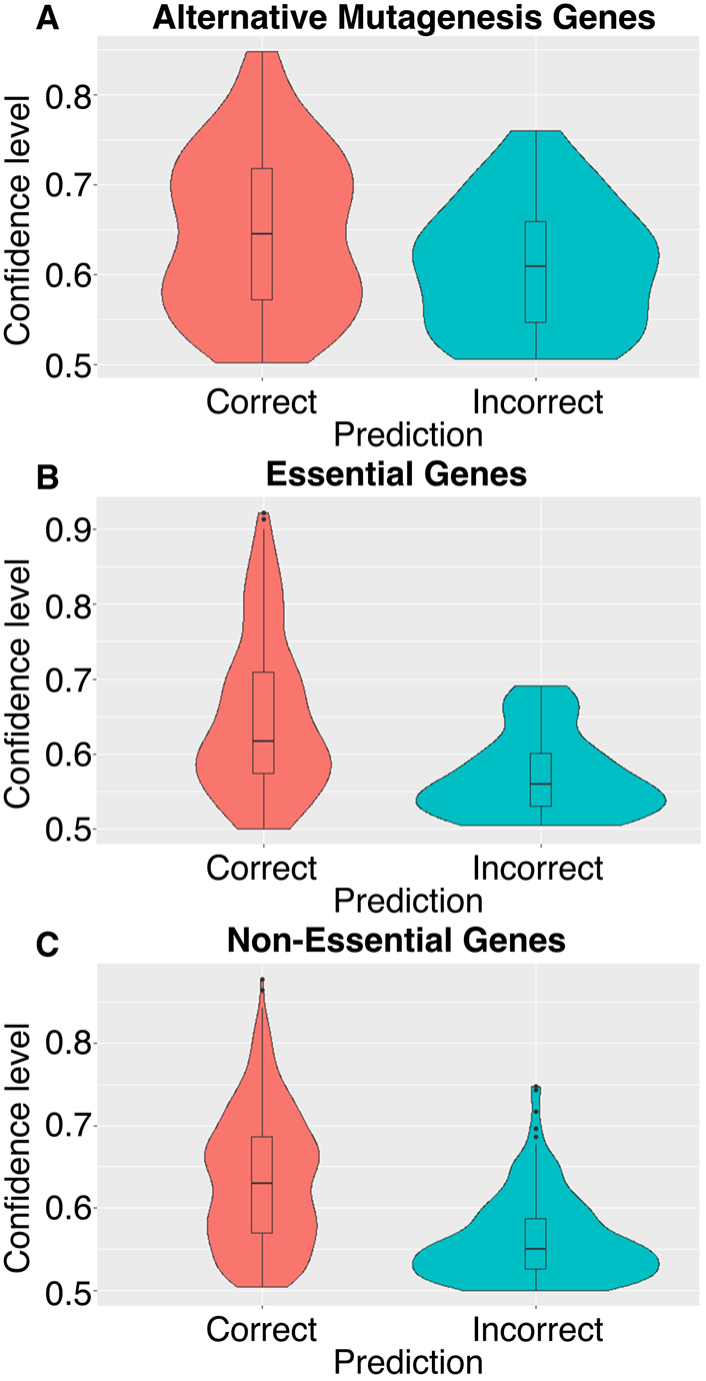
**Differences in ‘Essentiality’ gene prediction confidence levels for experimentally validated blind and alternative mutagenesis mouse genes.** (A-C) A Normal distribution was confirmed for alternative mutagenesis data (*n*=115 genes) using Shapiro–Wilk test. Welch's 2-sample *t*-test identified a significant difference between correct and incorrect prediction confidence-levels (*P*=0.0166) for predictions of alternative mutagenesis genes (A). Both essential (*n*=229 genes) and non-essential (*n*=802 genes) blind test set 1 data were not normally distributed (Shapiro–Wilk test). Using Wilcoxon's Rank-Sum 2-sided test, significant differences were found between prediction confidence levels of correct and incorrect predictions for essential (B) and non-essential (C) blind test set 1 genes (*P*=1.75×10^−7^ and *P*≤2.2×10^−16^, respectively).

We compared the accuracy of our predictions with experimental data generated by alternative mouse mutagenesis methodologies aside from targeted gene deletions. Data were collected from the MGI database (version 6.07) (Bult et al., 2016), using the search terms ‘Viable’ and ‘Lethal’ and specifying ‘Null/Knockout alleles’, with all chromosomes and generation methods selected other than ‘Targeted’, ‘Transgenic’ and ‘QTL’. We excluded targeted alleles because these are already in our training sets. We excluded transgenic alleles as some of these experiments assess overexpression or misexpression of genes, which are not directly comparable to the null alleles contained in our training sets. Finally, we excluded QTL alleles because these are not single gene effects. The search returned 201 essential genes and 29 non-essential genes. Duplicate entries, genes included in our test sets or genes found in our training sets were excluded from the analysis. Some genes were retrieved from both the essential and non-essential searches; these genes were categorised as either essential or non-essential following consultation of published phenotypes. Our final alternative mutagenesis method dataset included 116 essential and non-essential query genes, with allele types of ‘Gene trapped’, ‘Transposon induced’, ‘Chemically induced’, ‘Spontaneous’ or ‘Endonuclease mediated’, which were checked against our classifier predictions (Table S3). In 72% of cases, the essentiality classifier predicted the correct essentiality of the query genes, with 32 out of 116 genes being incorrectly predicted. The average prediction confidence level for incorrectly predicted genes was 0.608, with the mean confidence level for correct predictions being 0.647 (Fig. 3A). The difference in confidence levels between correct and incorrect predictions was significant (Welch's 2-sample *t*-test, *P*=0.0166), confirming that incorrectly predicted genes had lower confidence predictions and correctly predicted genes had higher prediction confidence levels. We also compared the prediction confidence for Test set 1 genes, and found a similar trend within both the essential and non-essential gene predictions, such that incorrect predictions were of significantly lower confidence than correct predictions (Fig. 3B,C). Thus, we conclude that our classifier predicts essentialities of genes that have been experimentally determined by mutagenesis methods other than targeted deletions, with greater than 72% of essentiality predictions correctly validated. The confidence levels of our predictions reflect their probable accuracy for all datasets examined.

Additionally, a recent publication listed mouse essential genes revealed from experiments to generate a haploid mouse embryonic stem cell biobank (Elling et al., 2017). A total of 23 essential genes were identified through experimental analysis as essential for mouse embryonic stem cell survival. Of these genes, 16 were contained within our prediction dataset. Our classifier accurately predicted the essentiality status for 15 of the 16 genes (94%; Table S6), demonstrating further successful application of our classifier to additional experimental data types.

### Enriched features of essential and non-essential genes

To understand the biological functions specific to essential and non-essential genes, we performed functional annotation of known and predicted essential and non-essential mouse genes using 4 distinct web tools to identify enriched features: Database for Annotation, Visualisation and Integrated Discovery (DAVID) v6.8 (Dennis et al., 2003), WebGestalt (2017 update) (Zhang et al., 2005), g:Profiler (2016 update) (Reimand et al., 2007) and PANTHER (v11.1) (Mi et al., 2016). Because our predicted gene datasets are considerably larger than the training sets we have previously analysed, we wished to explore whether or not the functional annotations of the predicted genes were similar to those of the genes with known essentiality status. Consistent with our previous work on experimentally validated mouse essential genes (Kabir et al., 2017), proteins encoded by predicted essential genes were found to be significantly enriched in localisation to intracellular locations, with 50.5% of genes annotated with the cellular component (CC) GO term ‘nucleus’. Furthermore, biological process (BP) and molecular function (MF) GO terms relating to translation, chromosome segregation, information processing, RNA splicing, mRNA processing and numerous metabolic process were commonly enriched in predicted essential or non-essential mouse genes (Table 1). Helicase protein domains and helicase-related terms were also frequently significantly enriched (*P*<0.05) in all webtool outputs for predicted essential genes. These results confirm that essential genes tend to have critical functions in DNA replication, DNA repair, transcription and translation, as helicases are known to be involved in these processes (Sedman et al., 2000). Disease pathways were frequently enriched amongst essential genes, including many cancers, and Huntington's, Alzheimer's and Parkinson's diseases, confirming prior reports that essential genes are disease related (Dickerson et al., 2011).

**Table 1.**
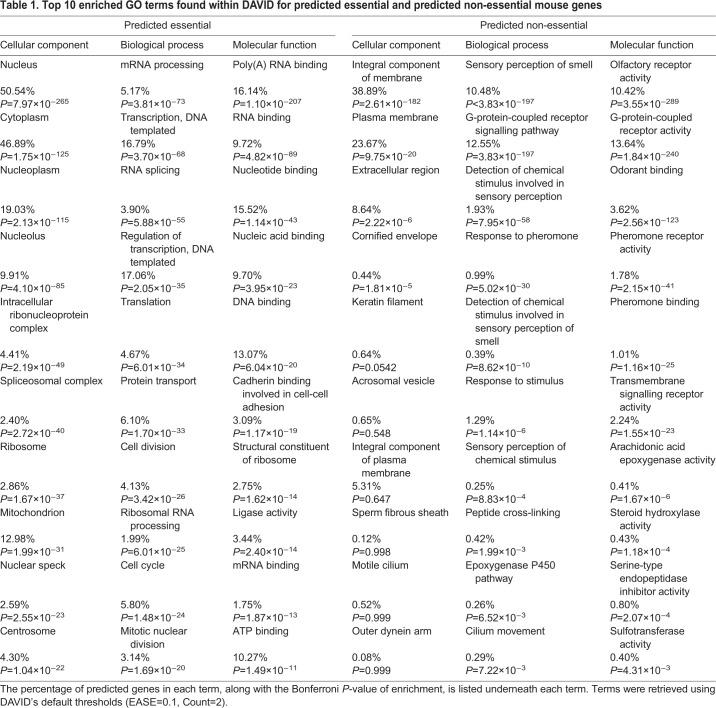
**Top 10 enriched GO terms found within DAVID for predicted essential and predicted non-essential mouse genes**

Conversely, the UniProt keywords ‘transmembrane helix’ and ‘transmembrane’ were significantly enriched in the predicted non-essential genes (*P*=1.10×10^−154^ and *P*=2.62×10^−154^, respectively), which is consistent with the significant enrichment of transmembrane proteins found in the known viable mouse genes previously examined (Kabir et al., 2017). Notably, the number of protein transmembrane domains was not a feature that was included in classifier training following GA feature selection, so it is interesting that this feature is prominent amongst the predicted non-essential genes even though it was not used in the classification criteria. We noted that olfactory functions were enriched in the predicted viable gene set, most likely due to the large number of olfactory receptor genes found in the mouse genome. We therefore excluded the olfactory receptors from our predicted viable gene dataset and performed the functional annotation analysis again to identify other features that are enriched once olfactory functions are excluded (Table S7).

Our findings on the functional enrichments of the large predicted gene datasets are consistent with the functions enriched in the smaller training datasets (Kabir et al., 2017), and can therefore identify biological requirements during development and postnatal life. Our classifier did not incorporate GO functional annotations within its selection criteria, so it is striking that there is consistent agreement between the GO functions enriched in genes with known essentiality status and genes with predicted essentiality status. In general, the known and predicted genes of either essentiality category share the same GO Slim annotations for BP, CC, MF and PANTHER protein domains, with deviation from the overall genome distribution for these annotations (Table 2). These findings highlight the functional differences between essential and non-essential genes.

**Table 2.**
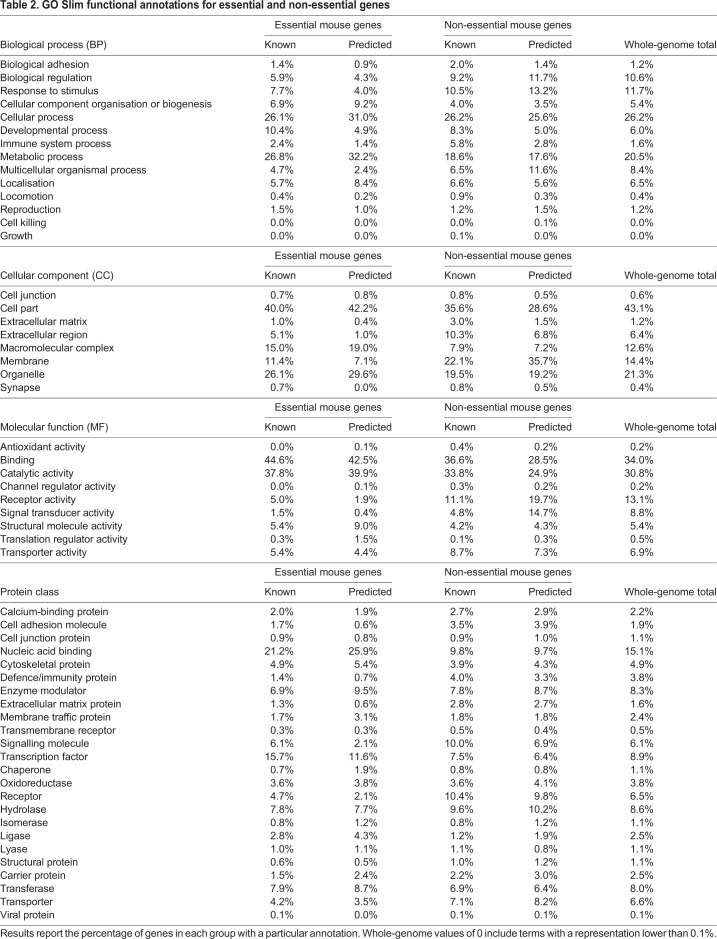
**GO Slim functional annotations for essential and non-essential genes**

### PPI networks of essential and non-essential genes

Since we found protein network features to be highly informative in our classifier, we sought to examine the protein network topology of predicted essential and non-essential genes for comparison with their known essentiality counterparts. All PPI network graphs can be represented by a scale-free model (Vella et al., 2017), as shown by the degree distribution of the networks, which fits a power-law curve (Fig. S1). In scale-free models, the degree value (i.e. number of interactions per network node) of most nodes is far from the mean. Only a few nodes in each network have a high number of interactions. However, PPIs of the essential genes datasets (known and predicted) form networks that are denser, having a higher average number of neighbours, a higher tendency to form clusters and less heterogeneity than the corresponding datasets of non-essential genes (Table 3), using network parameters as defined in Hubba (Lin et al., 2008; Dong and Horvath, 2007) and NetworkAnalyzer (Doncheva et al., 2012). We infer from the graph data that the PPI network generated from proteins encoded by essential genes shows higher connectivity than networks generated from non-essential genes, and that essential proteins are more likely to form hubs in the network (Table S8). Network features such as degree do differ between the known and predicted networks of both essentiality classes, indicating that the expectation that known and predicted proteins of a particular essentiality class will have the same properties could be an oversimplification.

**Table 3.**
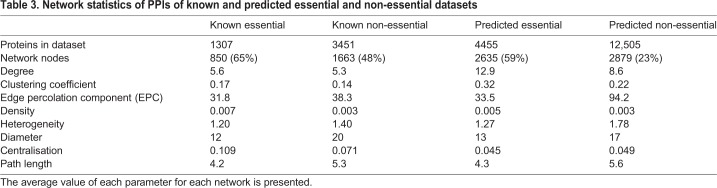
**Network statistics of PPIs of known and predicted essential and non-essential datasets**

### Chromosomal distribution of essential and non-essential genes

**Fig. 4.**
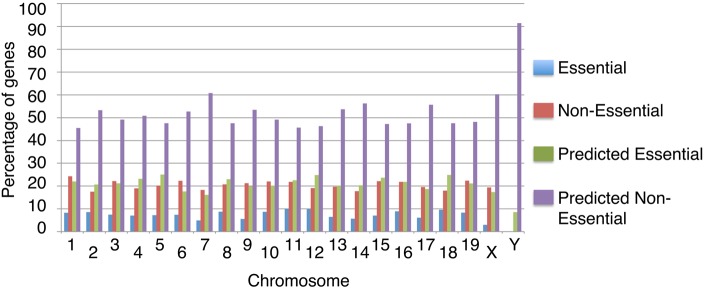
**The genomic distribution of essential and non-essential mouse genes, separated into known and predicted essentiality.** The percentages of essential and non-essential genes on each chromosome are compiled from the MED database. In the genome as a whole, we calculate that there are 28% essential genes and 72% non-essential genes when known and predicted essentiality statuses are combined. Data are provided in Table S8.

We examined the distribution of essential and non-essential genes within the mouse genome, partitioned by known and predicted essentiality status (Fig. 4; Table S9). Chromosomes 11, 12 and 18 have the highest proportion of known essential genes, which comprise 9.96%, 9.84% and 9.60% of their entire chromosomal gene content, respectively. Chromosomes 5, 12 and 18 have the highest proportions of predicted essential genes across the whole genome. This finding agrees with previous experimental work, including a balancer chromosome random chemical mutagenesis study that found that ∼60% of mutant phenotypes mapped to mouse Chromosome 11 were homozygous lethal (Kile et al., 2003), and an additional study that reported many embryonic lethal mutations map to mouse Chromosome 5 (Wilson et al., 2005).

The essential and non-essential training set and predicted gene lists were separately uploaded into the bioinformatics database DAVID v6.8 (Dennis et al., 2003), and significantly enriched chromosomes were identified in each dataset. In agreement with our genomic analysis, Chromosome 11 was significantly enriched for both known essential genes and predicted essential genes (Bonferroni-corrected *P*-values of 6.88×10^−5^ and 1.30×10^−3^, respectively). Chromosome 5 was the most significantly enriched chromosome in the predicted essential genes dataset, with 365 predicted essential genes (8.4% of 4329 genes) located on Chromosome 5 (Bonferroni-corrected *P*-value of 1.17×10^−3^). Similar results were obtained from WebGestalt (2017 update) (Table S10).

Chromosome 7 is the autosome with the highest combined percentage of known and predicted non-essential genes at over 79%. This result suggests that the majority of genes localised to this chromosome tend not to function in developmentally crucial processes. According to the DAVID functional annotation tool, Chromosome 7 was the most significantly enriched chromosome in the predicted non-essential genes dataset, with a Bonferroni corrected *P*-value of 3.10×10^−12^, containing 11.2% (1128 of 10,068 DAVID IDs) of predicted non-essential genes. Similar results were obtained with WebGestalt, finding 5 significantly over-represented (false discovery rate <0.05) cytogenetic bands belonging to Chromosome 7 for the predicted non-essential genes (Table S10). Three Chromosome 7 regions were also detected in the top 25 most significantly over-represented chromosomal locations for the known non-essential genes.

Overall, our findings show that there is variation in the distribution of essential and non-essential genes throughout the genome. These findings are consistent with a prior study on gene synteny and density, which found that Chromosome 7 contains far fewer essential genes than other mouse chromosomes, and that Chromosome 11 contains a high proportion of essential genes (Hentges et al., 2007). Additionally, experimental studies interrogating regions of mouse chromosomes through random chemical mutagenesis are consistent with our findings of gene essentiality predictions, indicating the localisation of essential genes on mouse Chromosomes 5 (Wilson et al., 2005) and 11 (Kile et al., 2003).

### Database of gene predictions

In order to facilitate searches for essential and non-essential genes, we created a database of mouse essentiality data (MED; http://essentiality.ls.manchester.ac.uk). The essentiality status of all protein-coding mouse genes, and the confidence level of essentiality predictions, is included in the MED database. The database has several search options, including gene symbol, MGI gene ID, Ensembl gene ID and chromosomal location. Additionally, lists of all essential or non-essential genes within the genome can be retrieved and downloaded, or lists of genes by essentiality status within a particular genomic region. The MED database should expedite searches for mouse gene essentiality status, based upon our criteria for essential gene identification (Kabir et al., 2017).

### Comparison to human essential and non-essential genes

We evaluated the applicability of our findings on mouse gene essentiality to human genes. We identified 1495 known human non-essential genes from the literature (Table S11) (MacArthur et al., 2012; Sulem et al., 2015; Kaiser et al., 2015; Saleheen et al., 2017). Manual identification of the mouse orthologues of these human genes was conducted using Homologene, Online Mendelian Inheritance in Man (OMIM), GeneCards and the UCSC Genome Browser. Following this, duplicate genes present in the data were removed, in addition to any read-through genes and non-RefSeq UCSC genes (as annotated in the UCSC genome browser). Human genes without known mouse orthologues were excluded from the analysis. We therefore identified 1260 known non-essential human genes with mouse orthologues. Known human essential genes were also collected from the literature, providing in total 5205 genes from 4 publications (Table S11) (Blomen et al., 2015; Lek et al., 2016; Shamseldin et al., 2015; Wang et al., 2015). As above, mouse orthologues of these essential human genes were identified, and read-through genes, duplicate genes and those without a mouse orthologue excluded from our analysis. We obtained a final dataset of 5084 essential human genes and their mouse equivalents.

We found that 337 of the 1260 human non-essential genes and 1811 of the 5084 human essential genes were contained within our mouse essential or non-essential training sets. We then assessed these human and mouse genes for matching essentiality (Table 4; Table S11) to determine whether a gene that is annotated as essential in humans is also known to be essential in the mouse. We found that 296 (87.83%) known non-essential human genes were found to be non-essential in mouse knockout experiments, with 41 (12.17%) essentiality mismatches (i.e. non-essential in human but essential in mouse). The 1811 known essential human genes had 956 (52.79%) essentiality matches to their mouse orthologues, leaving 855 (47.21%) essential human genes with mismatched essentialities with their mouse equivalent (Table S11). This discrepancy could reflect the physiological, biological and developmental differences between mouse and human. Essentiality mismatches could also be due to the methodology of identifying human essential genes, as 2 publications classified human essential genes as those that caused proliferation failure when knocked down in cell culture cancer lines (Wang et al., 2015; Blomen et al., 2015). Cell culture essential genes might not be required for whole-organism viability, and cancerous cells might require tumour-specific essential genes not essential for healthy cells (Guo et al., 2017). However, when only human essential genes identified by sequencing are compared with mouse essential genes, 54% of these genes have mismatched essentiality with their mouse orthologue (Table S11), suggesting that the methodology for essential gene identification does not play a significant role in explaining the divergent essentiality classifications. Differences in mouse and human physiology and selective pressures since the human-mouse evolutionary split (Thomas et al., 2012) could result in non-essential genes becoming essential and vice versa. Critically, most human studies are unable to be truly comparable to mouse studies due to inabilities to test human embryos experimentally. One study sequenced human embryonic DNA, yet was unable to unequivocally confirm that all mutated genes cause embryonic lethality (Shamseldin et al., 2015). Therefore, genes that are identified as essential in humans from experimental cell culture data or sequence analysis might not necessarily cause lethality during human development.

**Table 4.**
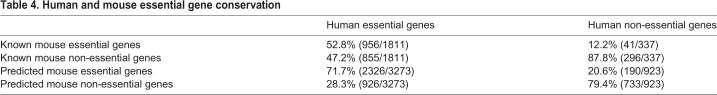
**Human and mouse essential gene conservation**

For the 923 non-essential human genes and 3273 essential human genes which were not contained in either mouse training set, our mouse classifier predictions had a high percentage of essentiality status matches (Table S11). For example, 71.1% (2326/3273) of the human essential genes were also predicted as being essential in mice. Additionally, 79.4% of the 923 human non-essential genes had the same essentiality prediction status as their mouse orthologues (Table S11). Some discrepancies between human and mouse gene essentiality status are expected due to biological differences, rather than inaccurate classifier performance, as it has been reported that at least 20% of shared human and mouse genes result in different phenotypes when functionally deleted (Liao and Zhang, 2008). These results therefore give confidence that our mouse gene predictions can be used to inform future mouse and human genetic research.

To discover whether features enriched in essential and non-essential mouse genes are also enriched in human genes of the same essentiality, the DAVID functional annotation tool was used to retrieve enriched annotations. Overall, enriched terms matched across both species: essential genes had DNA-binding, helicase, transcription and nucleus-related enrichment, with non-essential genes enriched in transport, receptor, signalling, immunity, and membrane and extracellular locations (Table S12). Information processing terms are therefore absolutely fundamental to all organisms for viability, survival and reproduction as they are found to be enriched in minimal gene sets of bacteria (Juhas et al., 2014), yeast (Acencio and Lemke, 2009), mouse and human (Yang et al., 2014). Inconsistencies included protein domains associated with ion channels being enriched in the human essential gene dataset, but also enriched in the mouse non-essential gene dataset. Ubiquitin-related and mRNA processing terms were enriched in human non-essential genes and also in mouse essential genes. This finding was unexpected, as ubiquitin and mRNA processing have key developmental functions (Tu et al., 2012; Vriend et al., 2015); therefore, discrepancies between mouse and human essentiality annotations might be due to reported human cellular essential genes not being essential at the organismal level.

## DISCUSSION

We compiled training sets from mouse knockout data to identify essential genes (Kabir et al., 2017), which were utilised to train several classifiers to predict gene essentiality. This work used a wide range of genomic features to predict essentiality, many of which have not been examined in previous studies (Yang et al., 2014). Our methodology has achieved greater 10-fold cross-validation classification accuracy than prior machine learning predictions of mouse knockout phenotypes (Yuan et al., 2012). Our classifier's performance is also more accurate than a support vector machine human essential gene classifier examined in jackknife tests and by 10-fold cross-validation (Yang et al., 2014). A strength of our study is the use of 2 blind test sets to further interrogate the validity of our classifier, which differs from other prior research generating mammalian essential gene classifiers (Yang et al., 2014; Yuan et al., 2012), but is similar to methodology utilised in a study to predict plant gene essentiality (Lloyd et al., 2015). The high accuracy of our predictions on the blind test sets, and the strong correlation between the confidence of our predictions and their accuracy, indicates that our classifier is discriminating between essential and non-essential genes. The percentage of genes predicted to be essential in the mouse genome using our classifier is similar to the percentage of genes found to be essential in mouse knockout experimental studies, and the properties we found to be enriched in mouse predicted essential genes are consistent with annotations of known mouse essential genes (White et al., 2013; Dickinson et al., 2016). Notably, we found that ∼20% of genes in our essential gene training dataset had been designated as non-essential genes by the IMPC (Koscielny et al., 2013). Although clearly the IMPC alleles produced viable mice, the majority of these genes had additional experimentally generated alleles reported in the MGI database that displayed lethal phenotypes. The IMPC database only contains reports of alleles generated as part of the IMPC project and not prior experimental data from other laboratories, which presents a limitation for utilising the IMPC data alone in determining the essentiality status of a given gene. The comparison of the MGI and IMPC datasets allows a quantification of the variation in experimental results for essentiality phenotypes that can be obtained from mouse knockout studies.

The 10 most informative features used in the random forest classifier to predict gene essentiality status relate to protein interactions or protein composition (Table S4). A study on human essential genes reported that topological properties of the PPI network are highly informative for predicting essential genes (Yang et al., 2014), and several studies on other organisms also find that protein interaction network features are useful for distinguishing essential and non-essential genes (Acencio and Lemke, 2009; Lloyd et al., 2015; Hwang et al., 2009; Li et al., 2014). In many species, essential genes occupy hubs within protein interaction networks (Lee et al., 2010; Liang and Li, 2007; Hwang et al., 2009); thus, it is understandable that protein network features are highly informative for predicting the essentiality of a gene with unknown essentiality status. Seven features reporting developmental gene expression levels are also highly discriminatory, because genes that are not expressed during development are unlikely to be essential for survival throughout gestation. Subcellular localisation features such as nucleus and plasma membrane were also found to have high information gain, which correlates with our finding that these same features showed significant statistical differences in their distribution amongst our training set genes (Kabir et al., 2017).

A publically available online database has been created to disseminate the essentiality predictions of mouse genes lacking experimental essentiality annotations (http://essentiality.ls.manchester.ac.uk), which is searchable by multiple identifiers and can produce lists of gene essentiality for download. We believe that our mouse gene essentiality status predictions will be useful for researchers seeking to create mouse mutants (a rapidly expanding area of biological research due to genome editing technology) (Singh et al., 2015), because researchers can quickly determine whether their gene of interest is likely to be essential or not. Owing to the conservation of function and essentiality status between mouse and human genes, knowledge of mouse gene essentiality will aid clinical geneticists seeking to interpret the impact of genome sequence variants on phenotype, a need that is rapidly increasing with the expanding use of genome and exome sequencing in clinical diagnostics. Knowledge of the composite set of essential genes of an organism is also of benefit for synthetic biology (Rancati et al., 2018).

Upon comparing our predictions of mouse gene essentiality with human gene essentiality annotations, we found a high degree of correlation between predicted mouse non-essential and essential genes and their human orthologues with known essentiality status. Similarly, we found a strong correlation between experimentally identified mouse non-essential genes and human known non-essential genes. Larger discrepancies were found between mouse known essential genes and human known essential genes, however, which we propose is related to the differing methodologies used to identify mouse and human essential genes, a hypothesis noted by others (Bartha et al., 2018). Given the prominence of mouse models for the study of human diseases (Rosenthal and Brown, 2007), an increased understanding of whether discrepancies in gene essentiality between these species represent biological differences or functional annotation differences will improve the interpretation of mouse model data.

## MATERIALS AND METHODS

### Compilation of datasets

Our essential and non-essential mouse gene datasets have previously been described (Kabir et al., 2017). We defined an essential gene as a gene causing lethality prior to postnatal day 3 in a single gene knockout experiment. Only single gene knockout (targeted deletion) experiments were considered. If a gene had a lethal phenotype in any knockout experiment, it was considered lethal, even if knockouts of other exons or on other strain backgrounds, or mutations generated by methods other than targeted deletion, did not have a lethal phenotype. IMPC data were retrieved through the ‘phenotypes’ query on the IMPC website (Koscielny et al., 2013), using the keywords ‘embryonic lethality’ for essential genes and MP keyword terms previously chosen for MGI searches (Kabir et al., 2017) for non-essential genes. IMPC subviable genes were obtained from the Embryo Development Special Report accessed on their website (Koscielny et al., 2013).

Alternative mouse mutagenesis-methodology data were collected from the MGI database. MGI genes were filtered using terms ‘Viable’ and ‘Lethal’ and specifying ‘Null/Knockout alleles’, with all chromosomes and generation methods selected, apart from ‘Targeted’, ‘Transgenic’ and ‘QTL’. Publications for genes retrieved with both ‘viable’ and ‘lethal’ keywords were manually assessed, allowing verification of genes as essential or non-essential. Duplicate genes and those in training sets were excluded. Genes essential in mouse embryonic stem cells were identified from the literature (Elling et al., 2017).

Human essential genes (Blomen et al., 2015; Shamseldin et al., 2015; Wang et al., 2015; Lek et al., 2016) and non-essential genes (MacArthur et al., 2012; Kaiser et al., 2015; Sulem et al., 2015; Saleheen et al., 2017) were retrieved from the literature. To compare human gene lists with mouse datasets, mouse orthologues were manually retrieved from OMIM (Amberger et al., 2015), HomoloGene at NCBI (NCBI Resource Coordinators, 2016), GeneCards (v4.4.1) (Stelzer et al., 2016) and the UCSC Genome Browser (Casper et al., 2017). Duplicate genes were excluded, as were read-through genes and non-RefSeq UCSC genes [as annotated in the UCSC genome browser (Casper et al., 2017)], along with human genes without mouse orthologues.

### Retrieval of gene features

Features including ‘gene length’, ‘transcript count’, ‘exon count’ and ‘transcript per million’ were computed based on data retrieved from Ensembl BioMart (Yates et al., 2016) and UniGene (Pontius et al., 2003; Stanton et al., 2003). The other genomic and protein-sequence-based features were retrieved directly from Ensembl (Cunningham et al., 2015), UniProt (UniProt Consortium, 2015), Pepstats (Rice et al., 2000) and SignalP (Petersen et al., 2011; UniProt Consortium, 2015). Mouse PPI data were obtained from the I2D database (Brown and Jurisica, 2005). In-depth descriptions of the features collected have previously been described (Kabir et al., 2017).

### Dataset balancing

Because the essential and non-essential mouse gene training sets differed in the number of genes, random subsampling with no replacement (Vitter, 1985) was used to select a class-balanced subset from the training data set with no duplicate instances in the subset.

### Discretisation

Discretisation (Han et al., 2011; Witten et al., 2016, 2011) of the numeric features of the training dataset was performed using the ChiMerge algorithm (Kerber, 1992) to remove noise and improve the speed of classifier training. Two adjacent intervals of each feature were merged into bigger intervals repeatedly, based on the chi-squared correlation of the 2 adjacent intervals and the class attribute. Initially, for each numeric value of a feature, an interval was created to contain the numeric value only. Then, a chi-squared test was used to test the hypothesis that the class attribute is independent of the 2 adjacent intervals. If the test was independent of the 2 adjacent intervals, they were merged; otherwise, they remained separate. Merging all pairs of adjacent intervals continued until the chi-squared value of every pair of adjacent intervals was greater than the chi-squared value determined with a significance level of 0.95.

### Machine learning classifiers

In this study, the mammalian essential gene prediction problem was formulated as a supervised binary classification problem. Given a mouse gene *p*, we intended to predict the corresponding class *y*, such that *p*∈*y* (Chen et al., 2012). We used Weka (version 3.6), a publicly available Java-based machine learning software (Hall et al., 2009), to implement the predictive classifier. We used naїve Bayes (Rish, 2001), J48 decision tree (Breiman et al., 1984), SVM (Cortes and Vapnik, 1995), logistic regression and Random Forest (Breiman, 2001) methods implemented in Weka as classifiers. Classifiers were trained on a fixed number of mouse genes labelled as essential or non-essential, each consisting of *m* features. Separate test datasets were also created that have not been included in the training datasets. We implemented 10-fold cross-validation on the training sets to assess the performance of each classifier, followed by 10-fold cross-validation on Test sets 1 and 2. Calculating the proportion of correctly predicted genes in these test datasets validated the performance of classifiers.

For the RBF kernel SVM, we set C to 50 and experimented with different values of gamma: 0.1, 0.05, 0.01, 0.005, 0.001, 0.0005 and 0.0001. For RBF kernel SVM and polynomial SVM, C is set to 50 because 50 is a common value for cost. For gamma, we tested the values 0.1, 0.05, 0.01, 0.005, 0.001, 0.0005 and 0.0001 to find the best value. Similarly, we tested polynonmial orders of 1, 2, 3 and 4. Polynomial kernel SVMs with the penalty term C of 50 and different orders 1, 2, 3 and 4 were trained using 10-fold cross-validation. For regularisation of logistic regression (LR), we used the default setting that the regulariser is set to w^2^ and the ridge (penalty term) is set to 10^−8^, where w is the weight vector of the LR. The default setting is the most common setting for LR. We treated the categorical features (e.g. subcellular localisations and types of proteins) of the gene essentiality dataset as numeric features and coded the discrete features as integers. For decision trees, we used the default parameter settings such that the confidence factor is set to the default value 0.25 (the confidence factor is used for pruning), and used the default C4.5 pruning instead of reduced error pruning. For naïve Bayes classifier, we assumed that the distribution of each attribute is Gaussian and used the probability density estimation to compute the prior probabilities. We used Bayes theorem to compute conditional probabilities.

### Performance measures

Classifier performance was evaluated by 10-fold cross-validation analysis, where each training dataset was randomly partitioned into 10 equal parts with 9 parts being used for model training and the remaining part used for testing. We used the cross-validation method to limit overfitting of the classifier.

The performance of each classifier was determined from the total number of essential genes predicted correctly (TP), essential genes predicted incorrectly (FN), non-essential genes predicted correctly (TN) and non-essential genes predicted incorrectly (FP), presented as a confusion matrix. From the counts of each of these, 3 performance measures, including the true-positive rate (recall or sensitivity; TPR), false-positive rate (FPR) and the overall classification accuracy, as defined by the following equations, were estimated:(1)
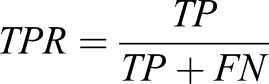
(2)
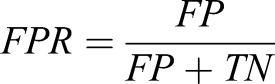
(3)
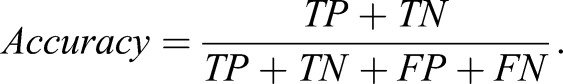


Further evaluation of classifier performance was achieved through the use of ROC curves, which were generated by plotting the TPRs against the FPRs at various threshold settings to present the probability of predicting true positives as a function of the probability of predicting false positives (Huang and Ling, 2005). The AUC of the ROC curves was used to estimate the overall prediction performance of the classifier, whereby an AUC of 1 represents a perfect prediction and an AUC of 0.5 represents a random guess.

### Feature selection algorithms

Feature selection was performed using the GA implemented in Weka. This wrapper method relies on a fitness function, population size, crossover probability, mutation probability and maximum number of generations to select relevant features in relation to the chosen classifier. The fitness function, generally defined as the accuracy of the chosen classifier, measured the quality of the solution. We used the Information Gain feature selection filter in Weka, which selects a subset of features from the pool of all features (Han et al., 2011) to estimate the worth (rank) of a feature by measuring its information gain with respect to a classification target. We did not examine all possible combinations of features, but ranked the features individually in order of significance to identify the most informative features for classification.

### Protein interaction network analysis

Four datasets of protein IDs corresponding to (1) known essential genes, (2) predicted essential genes, (3) known non-essential genes and (4) predicted non-essential genes were used to query the STRING database (Jensen et al., 2009) for PPIs. We used the stringApp (v.1.1.0) (Szklarczyk et al., 2017) plugin of Cytoscape (v.3.5.1) (Cline et al., 2007) to retrieve data from the STRING database. We filtered out PPIs for which there is no experimental evidence and those with a confidence score <0.4. Statistical analysis of the resulting networks was conducted using NetworkAnalyser (v.3.3.2) (Doncheva et al., 2012; Assenov et al., 2008) and the Cytoscape plugin cytoHubba (Chin et al., 2014). Unlinked nodes were eliminated prior to network analysis.

### Functional classification and annotation of gene sets

Four web-based applications – DAVID (v6.8) (Dennis et al., 2003), WebGestalt (2017 update) (Zhang et al., 2005), g:Profiler (Reimand et al., 2007) and PANTHER (v11.1) (Mi et al., 2016) – were used for functional evaluation of predicted and known genes, all utilising a *Mus musculus* genomic background. For each tool, 4 mouse gene sets were separately uploaded: (1) known essential genes, (2) predicted essential genes, (3) known non-essential genes and (4) predicted non-essential genes.

DAVID's functional annotation tool was employed, applying default thresholds (unless otherwise stated in results). Enrichment data were collected from DAVID's ‘Tissue Expression’, ‘UP_Keywords’, ‘Chromosome’, ‘KEGG_Pathway’, ‘InterPro’, Pfam’, ‘BioGrid’, ‘GOterm_BP_Direct’, ‘GOterm_CC_Direct’ and ‘GOterm_MF_Direct’ categories, and the top 50 results were analysed for each dataset. DAVID’s ‘Related Term’ tool was implemented, alongside biological knowledge, to place similar terms in groups.

WebGestalt's Over-Representation enrichment Analysis (ORA) tool was utilised (Zhang et al., 2005). Data for the top 25 most significant results for GO BP, CC and MF non-redundant terms, chromosomal location, Wiki and Panther Pathways, and Phenotype were retrieved. For g:Profiler (Reimand et al., 2007), Kyoto Encyclopedia of Genes and Genomes (KEGG) pathways, and mouse sequence homologs of the Human Phenotype Ontology and GO BP, CC and MF terms were retrieved.

Statistical over-representation was retrieved from PANTHER (Mi et al., 2016) for PANTHER Protein Classes, PANTHER Pathways, GO BP complete, GO CC complete and GO MF complete categories. Results were manually analysed, and terms over-represented in one essentiality and under-represented in either opposing essentiality gene-set were identified as differentiating terms. Additionally, PANTHER and WebGestalt provided visual and text-based GO Slim tools for functional classification of each dataset. GO Slim pie charts representing the whole mouse genome and our selected gene sets were generated from PANTHER, allowing comparative analysis. GO annotations from DAVID, WebGestalt and g:Profiler were combined to identify common significant GO terms enriched across multiple outputs.

Functional annotation for reported essential and non-essential human genes was completed using gene Ensembl IDs uploaded to DAVID. Six gene sets were separately uploaded: (1) essential human genes, (2) non-essential human genes, (3) essential mouse genes, (4) non-essential mouse genes, (5) ‘matching essentiality’ essential human genes, and (6) ‘matching essentiality’ non-essential human genes. A *Homo sapiens* background was applied for human gene lists and annotation results were retrieved from the same categories as stated above for mouse genes.

### Genomic distribution of essential and non-essential genes

Utilising the MED (http://essentiality.ls.manchester.ac.uk), the total number of genes on each mouse chromosome was retrieved, along with each gene's known or predicted essentiality. Genomic distribution analysis of essential and non-essential genes within the entire mouse genome, partitioned into known and predicted essentiality, was performed, and proportions of lethal and viable genes on each chromosome were determined. Chromosomal location and cytogenetic band enrichment for mouse essential and non-essential genes was identified from WebGestalt and DAVID.

### Essentiality model testing

Gene predictions were compared against blind and alternative mouse mutagenesis genes, both with currently validated essentialities, by testing known genes against their equivalent gene's predicted essentiality. Custom-written Python scripts (available on request) compared collated gene lists with model gene predictions.

### Statistics

All statistical analyses were carried out using R statistical software (R 3.0.1, The R Foundation for Statistical Computing). For all database functional analyses, the Bonferroni correction was applied to retrieve significantly enriched terms, with a statistical significance threshold of *P*<0.05 (unless otherwise stated). Distributions of plotted data were tested for normality using the Shapiro–Wilk test. For normally distributed data, Welch's 2-sided *t*-test for unequal variance was implemented, whereas for non-normally distributed data, the 2-sided non-parametric Wilcoxon Rank-Sum test was used, to determine statistical significance.

## Supplementary Material

Supplementary information
