# Supplementary material for: Identifying mouse developmental essential genes using machine learning
Source: Dis Model Mech. 2018 Dec 13;11(12):dmm034546. doi: 10.1242/dmm.034546 (PMC6307915; doi:10.1242/dmm.034546)
Supplement: Supplementary information [file dmm-11-034546-s1.pdf]

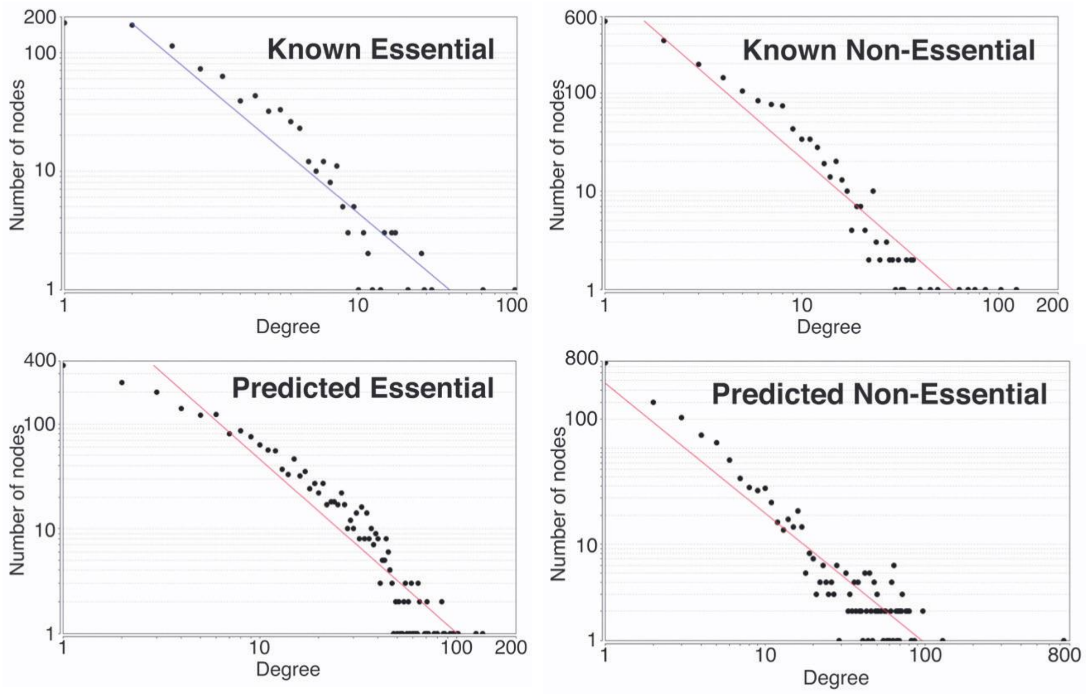

Fig S1. Node Degree Distribution. All networks exhibit a scale-free power law distribution of network nodes in the form **$y = ax^b$**. The dataset is labeled on each graph.

Table S1. Types of Features Collected for Mouse Genes.

Feature Type (number of features of that type)	Data Source
Gene Sequence Features (6)	Ensembl (Cunningham et al., 2015)
Protein Sequence Features (9)	Pepstats (McWilliam et al., 2013)
Amino Acid Composition (20)	Pepstats (McWilliam et al., 2013)
Post-translational Modification (5)	UniProt, SignalP (Petersen et al., 2011, UniProt, 2015)
Enzyme Class (6)	UniProt (UniProt, 2015)
Predicted Subcellular Localization (9)	WoLF PSORT (Horton et al., 2007)
Subcellular Localization (12)	UniProt (UniProt, 2015)
Transmembrane Count (1)	UniProt (UniProt, 2015)
PPI Network (20)	I2D, Cytoscape, Hubba (Chung-Yen Lin, 2008)
Gene Expression (13)	UniGene (Pontius JU, 2003)
Evolutionary Age (1)	Ensembl (Cunningham et al., 2015)

CHUNG-YEN LIN, C.-H. C., HSIN-HUNG WU, SHU-HWA CHEN, CHIN-WEN HO, AND MING-TAT KO 2008. Hubba: hub objects analyzer—a framework of interactome hubs identification for network biology. *Nucleic Acids Res.*, 36, W438-W443.

CUNNINGHAM, F., AMODE, M. R., BARRELL, D., BEAL, K., BILLIS, K., BRENT, S., CARVALHO-SILVA, D., CLAPHAM, P., COATES, G., FITZGERALD, S., GIL, L., GIRON, C. G., GORDON, L., HOURLIER, T., HUNT, S. E., JANACEK, S. H., JOHNSON, N., JUETTEMANN, T., KAHARI, A. K., KEENAN, S., MARTIN, F. J., MAUREL, T., MCLAREN, W., MURPHY, D. N., NAG, R., OVERDUIN, B., PARKER, A., PATRICIO, M., PERRY, E., PIGNATELLI, M., RIAT, H. S., SHEPPARD, D., TAYLOR, K., THORMANN, A., VULLO, A., WILDER, S. P., ZADISSA, A., AKEN, B. L., BIRNEY, E., HARROW, J., KINSELLA, R., MUFFATO,

Disease Models & Mechanisms • Supplementary information

M., RUFFIER, M., SEARLE, S. M., SPUDICH, G., TREVANION, S. J., YATES, A., ZERBINO, D. R. & FLICEK, P. 2015. Ensembl 2015. *Nucleic Acids Res,* 43**,** D662-9.

HORTON, P., PARK, K. J., OBAYASHI, T., FUJITA, N., HARADA, H., ADAMS-COLLIER, C. J. & NAKAI, K. 2007. WoLF PSORT: protein localization predictor. *Nucleic Acids Res,* 35**,** W585-7.

MCWILLIAM, H., LI, W., ULUDAG, M., SQUIZZATO, S., PARK, Y. M., BUSO, N., COWLEY, A. P. & LOPEZ, R. 2013. Analysis Tool Web Services from the EMBL-EBI. *Nucleic Acids Res,* 41**,** W597-600.

PETERSEN, T. N., BRUNAK, S., VON HEIJNE, G. & NIELSEN, H. 2011. SignalP 4.0: discriminating signal peptides from transmembrane regions. *Nat Methods,* 8**,** 785-6.

PONTIUS JU, W. L., SCHULER GD 2003. UniGene: a unified view of the transcriptome. *The NCBI Handbook.* Bethesda (MD): National Center for Biotechnology Information.

UNIPROT, C. 2015. UniProt: a hub for protein information. *Nucleic Acids Res,* 43**,** D204-12.

Table S2. Features collected.

Click here to Download Table S2

Table S3. Training set and test set genes and classifier predictions.

Click here to Download Table S3

Table S4. Classifier performance.

Click here to Download Table S4

Table S5. Comparison of phenotypes of our training set genes from MGI to IMPC lethal, subviable, and viable alleles.

Click here to Download Table S5

Table S6. Classifier prediction comparisons.

Click here to Download Table S6

Table S7. Enriched features of predicted viable gene set excluding olfactory receptor genes.

Click here to Download Table S7

Table S8. PPIs: interpretation of graph analysis results of essential (e) and non-essential (ne), known and predicted (pred) datasets.

Parameter	Network analysis	Inference
**Average number of neighbours (ANN)**	$ANN^{e}_{pred} > ANN^{e}_{known} > ANN^{ne}_{pred} > ANN^{ne}_{known}$	Higher average connectivity of the essential protein interaction networks (see also Density, which is a normalised version of this parameter)
**Clustering coefficient (CC)**	$CC^{e}_{pred} > CC^{ne}_{pred} > CC^{e}_{known} > CC^{ne}_{known}$	Higher degree of modular organisation of the essential PPI networks
**Diameter (DI)**	$DI^{ne}_{pred} > DI^{ne}_{known} > DI^{e}_{pred} > DI^{e}_{known}$	Higher degree of connectivity of the essential PPI networks
**Network heterogeneity (H)**	$H^{ne}_{pred} > H^{ne}_{known} > H^{e}_{pred} > H^{e}_{known}$	Higher tendency of the essential PPI networks to contain hub nodes
**Density (D)**	$D^{e}_{pred} > D^{e}_{known} > D^{ne}_{pred} = D^{ne}_{known}$	Higher degree of connectivity of the essential PPI networks
**Network centralisation (NC)**	$NC^{ne}_{pred} > NC^{ne}_{known} > NC^{e}_{pred} > NC^{e}_{known}$	Greater tendency of the non-essential PPI networks towards a star-like topology

Disease Models & Mechanisms • Supplementary information

Table S9. Genomic distribution of essential and non-essential genes.

Chromosome	Total gene number	% Essential	% Non-Essential	% Predicted Essential	% Predicted Non-Essential
**1**	1166	8.23	24.27	21.96	45.54
**2**	1783	8.52	17.50	20.64	53.33
**3**	965	7.46	22.18	21.14	49.22
**4**	1266	7.03	18.96	23.14	50.87
**5**	1239	7.18	20.18	25.02	47.62
**6**	1101	7.36	22.25	17.62	52.77
**7**	1914	4.86	18.23	16.09	60.82
**8**	1010	8.71	20.69	22.97	47.62
**9**	1193	5.53	21.21	19.78	53.48
**10**	969	8.67	21.98	20.12	49.22
**11**	1576	9.96	21.83	22.53	45.69
**12**	630	9.84	19.05	24.76	46.35
**13**	778	6.43	19.67	20.18	53.73
**14**	867	5.65	17.76	20.30	56.29
**15**	755	7.02	22.12	23.58	47.28
**16**	629	8.90	21.78	21.78	47.54
**17**	1014	6.11	19.53	18.64	55.72
**18**	479	9.60	17.95	24.84	47.60
**19**	672	8.33	22.32	21.13	48.21

Disease Models & Mechanisms • Supplementary information

**X**	872	2.98	19.38	17.32	60.32
**Y**	82	0	0	8.54	91.46
**Total genome**	20960	7.29	20.26	20.82	51.64

Table S10. Over-represented chromosomal regions.

Click here to Download Table S10

Table S11. Human essential and non-essential genes analysed.

Click here to Download Table S11

Table S12. Functional enrichment comparisons.

Click here to Download Table S12

Disease Models & Mechanisms • Supplementary information
